# Causes of delays in construction projects in the Province of Aceh, Indonesia

**DOI:** 10.1371/journal.pone.0263337

**Published:** 2022-01-28

**Authors:** Anita Rauzana, Wira Dharma

**Affiliations:** 1 Department of Civil Engineering, Faculty of Engineering, Universitas Syiah Kuala, Aceh, Indonesia; 2 Faculty of Mathematics and Natural Sciences, Universitas Syiah Kuala, Aceh, Indonesia; Szechenyi Istvan University: Szechenyi Istvan Egyetem, HUNGARY

## Abstract

Implementations of construction projects in Indonesia, especially in Aceh Province, are often delayed. Time, quality, and cost are three important components of planning a construction project. The benchmark for a successful construction project is the project completion time being in accordance with the time period specified in the contract. In project implementation, there is often a risk of delays in completing construction projects that can cause losses and fines; therefore, it is necessary to know the risk factors potentially causing project delays. The purpose of this study was to identify the risk factors causing delays greatly affecting construction projects in Aceh Province. The data used in this study were questionnaire data distributed to 68 respondents. The data processing methods included validity tests, reliability tests, and the construction of descriptive statistics. Ultimately, 60 delay factors were obtained; of these, 30 risk indicators were included in the *very influential* category with a mode value of (= 5), 29 delay risk indicators were in the *high influence* category with a mode value of (= 4), and one indicator was included in the category of *medium influence* (= 3).

## Introduction

The development of construction projects in Indonesia, especially in the province of Aceh, is growing rapidly, in line with the fulfilment of the basic needs of the community. Various types of risks can occur in any construction project. The greater the level of complexity of a project, the greater the risk that must be accepted. The risk will affect the level of productivity, project quality, time, and project costs. Risk can cause delays and losses; moreover, a risk is generally an unexpected event. The risk of delays in construction projects often occurs in developing countries [1]. Even when activities have been planned as well as possible, uncertainty often remains. The risks occurring in construction projects cannot be eliminated, but can be reduced or transferred from one party to another [2].

A project delay is a risk that often occurs, and can cause losses during the implementation stage of a construction project. The main risk factors for delays are financial and economic problems, contractors who lack experience and expertise, consultants’ delays in supervising work, slow decision making, using the wrong consultants when issuing instructions, and lack of materials [3]. If there is a delay in one of the projects on a critical path, it will cause delays in the next work. As a result of this delay, the costs that had been estimated in the planning stage tend to undergo a larger change. Therefore, project delays are a major contributor to project cost overruns. The most influential risk factors for delays are socio-economic and cultural factors [4].

The risk of delays is caused by various factors, such as those concerning materials, equipment, financial, environmental, labour, planning, and management. To minimise the occurrence of delays in subsequent projects, every factor should be considered properly in the early stages of starting work, so that time risks can be prevented or avoided (along with the corresponding implementation costs). The purpose of this study is to identify the risk factors causing significantly influential delays on construction projects in Aceh Province. Construction projects are dynamic and risky. Risk can affect the productivity, performance level, quality, time, and cost of a project. Risk is an unexpected event. Even when an activity has been planned as well as possible, uncertainty remains [2]. Project time delays often occur and can cause losses in construction projects [4–6]. Construction projects often experience delays of up to 80% [7].

Risk is an unexpected event, and negatively affects a project as a result of uncertainty. Risk is associated with the possibility or probability of occurrence of expected events. Risk is a factor that cannot be expected to cause loss, damage, or loss [8]. The risks in a project can cause losses, and an inability to meet the planned project objectives [9]. A risk has two main components for one event, namely, the probability of the event, and the impact of the event that occurs. The risk of a project can be considered as the cumulative impact of uncertainties resulting in the failure to achieve project targets, namely in regards to costs, time, quality, and work.

Previous research has shown that delays in project schedules are frequently owing to the swelling of costs [10]. Project risks can cause losses in construction projects [11, 12] and increase the cost of construction; moreover, they can result in poor productivity on the project, legal issues, and termination of contracts [13]. The most influential causes of delays in construction are the change order and cost-estimated errors [14, 15].

The risk of delays in construction projects can lead to losses and delays in projects. Indicators of a project’s success include the on-time implementation of the construction project, along with costs, quality, and security. Implementations of construction projects often face various types of risks. One of the largest construction risks comprises delays in construction projects, and the risk of delays occurring in each construction project can vary [16]. Contracting companies are more significantly affected by project risks and delays relative to consultants, who are considered as the least affected. Many small and large qualified contractors in recent years have struggled to overcome the problems of risks in delays or cost overruns, mainly because contractors do not have the experience and ability to identify the important causes of delay risks occurring in the implementation of construction projects. The identification of the main variables causing delays by project managers can help them manage risks more adequately [17].

The risk of a delay arises in the inability to meet the planned completion time [18], i.e. a project completion time that has passed the schedule determined based on the contract documents or the time, or that has exceeded the schedule agreed upon by the parties involved in the project [19]. A delay is the period of time by which a project late or postponed [20]. The timely implementation of construction projects is the main parameter for measuring the success of a project [21]. Providing an effective time for completion is often the main goal of the project, i.e. to increase profits [5, 22].

Poor project schedule management can lead to delays and losses [22]. Project risk management is very important in overcoming losses and delays and in improving project performance, and an effective project time can increase profits for contractors. Project delays can be caused by change orders and poor cost estimates [14, 15]. The project execution time must be in accordance with the planning time in the contract, and if the contractor cannot complete the project in accordance with the time specified in the contract document, the contractor will be given a financial penalty. The contents of the contract document may also contain provisions for contractors to be granted an extension of time for certain other types of delays, such as inclement weather [23].

There are four types of risks known to exist: 1) operational risks, namely risks related to organisational operations, including risks covering organisational systems, activity processes, technology, and human resources; 2) financial risks, i.e. risks affecting the financial performance of an organisation, such as those occurring as a result of currency fluctuations, inflation, erratic interest rates, credit restrictions, liquidity risk, and changes in market conditions; 3) hazard risks, i.e. those concerning accident rates, damage caused by fires, earthquakes, physical threats, and others; and 4) strategic risks, including the risks concerning a company’s strategy, along with political conditions, economic conditions, and laws [24]. Uncertainty is an important issue when conducting a risk assessment [25].

The delay in government projects in Aceh Province is a "disease" that always appears at the end of every year in Aceh Province, Indonesia. Warnings or threats to terminate the contract are often made by the governor or deputy governor, but delays in project completion always occur. In project implementation, project delays and losses often occur, the contractor company does not specify and identify the factors causing project delays. Therefore, research is needed to identify and analyze the factors causing delays in construction projects, to avoid losses and cost overruns in project implementation. There are three main categories of causes of construction project delays, namely design errors, construction problems, and third-party problems [26]. construction project delays are caused by unclear or ambiguous job boundaries, inaccurate estimates, and the occurrence of price fluctuations [27]. Improper planning and supervision, and limited management experience can result in project delays and cost overruns [28, 29]. Further studies need to be carried out to identify and analyze the causes of delays in construction projects in Indonesia, especially in Aceh Province, given the country’s complex conditions, such as socio-cultural diversity, low education level, weak community economy, geographical location, social conflicts, politics, and the economic crisis.

## Methods

### Questionnaire design

The primary data used in this research were in the form of questionnaire data. The questionnaire data were collected by distributing questionnaires to 68 contractor companies. The questionnaires were distributed to respondents using a closed questionnaire, where the respondents only chose from provided answers provided. The questionnaire was divided into two parts: questionnaire A and questionnaire B.

1. Questionnaire A concerned the characteristics of respondents, including their gender, age, latest education, company qualifications, and experience in the field of building construction.
2. Questionnaire B concerned the levels of influence of risk factors on project delays. The measurement of the answers was based on a Likert scale where each respondent’s answer was expressed as an assessment, as shown in Table 1.

**Table 1:** Likert scale.

No.	Category	Score
1	Very high influence	5
2	High influence	4
3	Medium influence	3
4	Low influence	2
5	Very low influence	1

The research population was a contractor company domiciled in the Aceh Province. Based on data from the Construction Services Development Institute, the total number of contracting companies was 215. Furthermore, the number of samples was determined using the Slovin formula, where the sampling is based on the population [30].


$$\text{n}=\frac{N}{1+(N\;x\;e^{2})}$$ (1)

Slovin’s formula was used with an error rate of 10%. Based on the Slovin formula, 68 research samples were obtained. Probability sampling was also used in this study, and provided an opportunity for the population to become members of the sample, via random sampling in distributing the questionnaires [31]. The research data processing method in this research was based on Statistical Package for the Social Sciences. The data processing methods included tests, reliability tests, and descriptive analysis. This study was conducted in accordance with the regulations of the Institutional Review Board of Syiah Kuala University in Indonesia, and all participants gave their informed consent.

### Descriptive statistics

Descriptive analysis is a form of research data analysis used to test the generalisation of research results based on one sample [32]. A descriptive analysis uses one or more variables but is independent; therefore, this analysis is not in the form of comparisons or relationships. Descriptive statistics comprises the part of statistics that studies how to collect data and present data so that it is easy to understand. Descriptive statistics are only related to describing or providing information about data, situations, or phenomena. Descriptive statistics function to explain conditions, symptoms, or problems. Drawing conclusions on descriptive statistics (if any) is only aimed at the existing dataset. Based on the scope of the discussion, descriptive statistics can include frequency distributions, such as distribution graphs (histograms, frequency polygons), and measures of central values (mean, median, mode, quartiles) [32].

## Results and discussion

### Validity test

The results of this study show that all statement items on the questionnaire have a value of Rcount > Rtable, where the Rtable value is 0.239. Therefore, the validity tests conducted on all questions are all valid. Thus, the study continued with reliability testing.

### Reliability test

A reliability test was used in this study to test whether the variables in the questionnaire were reliable. The reliability test criteria were as follows: if the Cronbach Alpha value was > 0.6, then the variable in the questionnaire was reliable; if the Cronbach’s alpha value was < 0.6, then the variable was not reliable. The results from the reliability tests for each variable are shown in Table 2.

**Table 2:** Reliability test.

No.	Variable	Cronbach Alpha	Conclusion
1	Material (X_1_)	0,943	Reliable
2	Equipment (X_2_)	0,819	Reliable
3	Financial (X_3_)	0,916	Reliable
4	Environment (X_4_)	0,903	Reliable
5	Labour (X_5_)	0,944	Reliable
6	Implementation (X_6_)	0,787	Reliable
7	Management (X_7_)	0,937	Reliable
8	Political (X_8_)	0,638	Reliable
9	Criminal (X_9_)	0,913	Reliable
10	Project Manager (X_10_)	0,902	Reliable

Table 2 shows that all variables in the questionnaire have Cronbach Alpha > 0.6. Therefore, all variables can be considered as reliable.

### Characteristics of respondents

The characteristics of the 68 respondents, i.e. project managers from contractor companies in Aceh Province, are provided below. The purpose of stating the characteristics of respondents is to provide an overview regarding the conditions of the respondents comprising the samples in the study [33].

From Table 3, it can be concluded that the respondents were entirely male (100%), the age range of the respondents with the highest frequency was 31–40 years (60.29%), the highest proportion of respondent education level was for undergraduate (91.18%), the highest proportion of respondents’ company qualification was M1 qualification (92.65%), and most respondents’ experience levels in regards to construction projects was 3–5 years (60.29%).

**Table 3:** Characteristics of respondents.

No.	Characteristics of Respondents	Frequency	Percentage
1	Gender		
	a. Man	68	100.00%
	b. Woman	0	0.00%
	Total	68	100.00%
2	Age		
	a. 20–30 years	-	0.00%
	b. 31–40 years	41	60.29%
	c. 41–50 years	27	39.71%
	d. > 50 years	-	0.00%
	Total	68	100.00%
3	Graduates		
	a. High School/equivalent	-	0.00%
	b. Diploma	-	0.00%
	c. Bachelor	62	91.18%
	d. Master	6	8.82%
	e. Doctoral	-	0.00%
	Total	68	100.00%
4	Company qualification		
	a. M_1_	63	92.65%
	b. M_2_	4	5.88%
	c. B_1_	1	1.47%
	Total	68	100.00%
5	Long Experience in the construction field		
	a. 0–2 years	-	0.00%
	b. 3–5 years	41	60.29%
	c. 6–8 years	27	39.71%
	d. > 8 years	-	0.00%
	Total	68	100.00%

Table 4 shows that, the projects observed were construction projects that have been implemented starting from 2012–2020. Questionnaire data was distributed to contracting companies that have implemented projects starting from 2012–2020, with the source of funds coming from the Aceh Province Revenue and Expenditure Budget. Respondents in this study were contractor companies that have medium qualifications (M1, M2), and large qualifications (B1). M1 companies have a project cost limit of ≤ 10 billion Rp, M2 companies of ≤ 50 billion Rp, and B1 companies have a project cost limit of ≤ 250 billion Rp.

**Table 4:** Information of respondents.

Respondents	Qualifications	Numbers	Cost limit for one project (Rp)
M1	Medium	198	≤ 10 billion
M2	Medium	14	≤ 50 billion
B1	Large	3	≤ 250 billion
Number of population		215	

### Level of influence of the risk of delay

A descriptive statistical analysis was used to determine the frequency of the respondents’ characteristics, and to describe the level of influence of the risk factors for delays occurring during the implementation of construction projects in Aceh Province.

Based on Table 5, there were 30 risk indicators in the ‘very influential’ category with a mode value of (= 5), 29 indicators in the ‘high influence’ category with a mode value of (= 4), and one indicator including the ‘medium influence’ category (= 3). Based on the research results obtained, the 30 indicators in the category of ‘very influential’ are described in more detail below.

1. Material factors, including indicators of material changes in form, function, and specifications, material damages in storage, inaccuracies in ordering materials, inefficient use of materials, delayed material arrivals, and poor calculation of material requirements. Project delays are often caused by a lack of availability and/or supply of materials [3, 5]. The project resources that need to be considered are the materials, and project material management is needed to avoid waste and to provide performance in accordance with planning. Thus, project development activities need to be continuously carried out without experiencing significant obstacles.
2. Equipment factors, i.e. indicators of equipment breakdowns, shortages of equipment, and poor equipment productivity. The dominant cause of delays is damage to equipment and tools [5, 34]. In construction work, work delays often occur at a planned time. This happens because of the ineffective implementation of equipment management for the project work [10]. In every construction project implementation, tools become a very significant factor in determining the process of conducting the work properly, correctly, and smoothly. As such, when talking about construction projects, tools cannot be separated from the equipment for supporting the implementation of a project. Project equipment is used to assist and facilitate project implementation, and it is hoped that contractors can carry out equipment management properly so that projects can run effectively and efficiently and avoid project delays.
3. Financial factors, namely indicators of poor financial conditions during implementation, delays in the payment process by the owner, high operational costs, and overhead from contractors. In general, every project requires financing for initial cash to start its activities. Even if a project has an advanced payment facility, initial cash is still required. This is because the down payment given at the start of the work is not large. Inadequate financial availability from contractors disrupts smooth completion of a project. Managing project finances aims to meet project capital and to manage the obtained capital as well as possible. Financial problems can lead to project delays and losses [3, 35].
4. Labour factors, namely indicators of shortages of labour, labour incapacities, owner interventions, labour fatigue owing to overtime, labour absenteeism rates, and a lack of workforce motivation. Human resources are one of the most influential factors in project works, including construction works. A project work not supported by good human resources in terms of quality and effectiveness will not provide maximum and satisfactory results in a project. The inappropriate use of human resources can result in significant losses in construction projects. The main causes of project delays are a lack of motivation and poor worker skills, resulting in losses and delays [10, 36].
5. Implementation factors, namely design change indicators and design errors from planners that require additional work. Errors and incomplete designs in project implementation can lead to risks of project losses and delays. Design is a very important stage in project implementation; drawing and specification errors can result in increased project costs, poor quality and safety, and delays in execution time [37, 38].
6. Management factors, including indicators of errors in understanding contract documents, and errors in execution. Construction failures occur owing to, e.g. errors in project implementation methods and in understanding contract documents [21, 34]. These failures can be technical or non-technical failures. A construction work failure is a condition of the construction work that does not follow the work specifications as agreed in the construction work contract, either partially or wholly, as a result of the error of the service user or service provider.
7. Political factors, namely indicators regarding a lack of coordination between relevant agencies in decision-making affecting construction project work, inputs from other agencies resulting in changes to the design and technical work, delays in the approval of the budget, and rejections from certain mass organisations for the sake of the group’s interests. A frequent cause of project is a political factor, namely, intervention by political leaders [34, 39]. The possibility of political risk in the form of regulatory/policy changes in infrastructure projects is quite high. This is because infrastructure development involves many government agencies, including both central and regional governments.
8. Project manager factors, namely the poor ability of a project manager in selecting personnel involved in the project, lack of project manager experience in dividing tasks and responsibilities, and lack of project manager experience in scheduling all work activities. A lack of project management experience can affect project delays [40]. Inexperienced project managers can result in project delays with impacts on (i.e. increased) costs [40]. A successful project is achieved if the cost, time, and quality factors have been achieved. If one of them is not fulfilled, then the project is not fully successful. For this reason, a reliable and experienced project manager is required to meet the required competency requirements. The competence of a project manager can be measured based on knowledge, skills, and attitude [41].

**Table 5:** Level of influence of the risk of delay.

Code Item	Risk of delays	n	Score	Mode	Interpretation
**X1**	**Material**				
X1.1	Lack of construction materials	68	301	4	High influence
X1.2	Material changes in form, function and specifications	68	306	5	Very influential
X1.3	Material delivery delay	68	296	4	High influence
X1.4	Material damage in storage	68	311	5	Very influential
X1.5	Scarcity of materials	68	301	4	High influence
X1.6	Inaccuracy in ordering materials	68	287	5	Very influential
X1.7	Inefficient use of materials	68	306	5	Very influential
X1.8	Delayed material arrival	68	291	5	Very influential
X1.9	Poor calculation of material requirements	68	307	5	Very influential
**X2**	**Equipment**				
X2.1	Equipment breakdown	68	312	5	Very influential
X2.2	Lack of equipment	68	310	5	Very influential
X2.3	Poor equipment productivity	68	306	5	Very influential
**X3**	**Financial**				
X3.1	Poor financial condition during implementation	68	317	5	Very influential
X3.2	Delay in the payment process by the owner	68	319	5	Very influential
X3.3	No financial support from banks for additional working capital	68	296	4	High influence
X3.4	High operational costs and overhead by contractors	68	317	5	Very influential
**X4**	**Environment**				
X4.1	Effect of weather on construction activities	68	296	4	High influence
X4.2	Poor environmental safety	68	289	4	High influence
X4.3	Geological problems on site	68	298	4	High influence
X4.4	Lack of communication between contractors and the community	68	289	4	High influence
X4.5	Difficult access for heavy equipment to the project site	68	267	4	High influence
**X5**	**Labour**				
X5.1	Shortage of labour	68	285	5	Very influential
X5.2	Labour incapacity	68	294	5	Very influential
X5.3	Owner’s intervention	68	307	5	Very influential
X5.4	Labour fatigue owing to overtime	68	282	5	Very influential
X5.5	Lack of awareness of project workers on occupational safety and health	68	305	4	High influence
X5.6	Replacement of new workers	68	276	4	High influence
X5.7	Labour absenteeism rate	68	307	5	Very influential
X5.8	Lack of workforce motivation	68	305	5	Very influential
**X6**	**Implementation**				
X6.1	Licensing delay	68	259	3	Medium influence
X6.2	There is a design change	68	311	5	Very influential
X6.3	Design errors by planners	68	315	5	Very influential
X6.4	There is additional work	68	312	5	Very influential
**X7**	**Management**				
X7.1	Weak time control system	68	291	4	High influence
X7.2	The arrangement of the sequence of activities is not good	68	297	4	High influence
X7.3	No evaluation of job specifications before implementation	68	291	4	High influence
X7.4	There is no operating procedure for each job	68	301	4	High influence
X7.5	Error in understanding contract documents	68	306	5	Very influential
X7.6	Poor occupational health and safety management	68	300	4	High influence
X7.7	Improper quality management procedures	68	290	4	High influence
X7.8	Error in using execution method	68	303	5	Very influential
**X8**	**Political**				
X8.1	News from print and electronic media that are counterproductive to project implementation	68	234	4	High influence
X8.2	Lack of coordination between relevant agencies in decision making that can affect construction project work	68	302	5	Very influential
X8.3	Inputs from other agencies that result in changes to the design and technical work	68	313	5	Very influential
X8.4	The procedure for permitting the implementation of development that is complicated by various parties	68	297	4	High influence
X8.5	Delay in the approval of the budget	68	327	5	Very influential
X8.6	Rejection from certain mass organisations for the sake of the group’s interests	68	318	5	Very influential
**X9**	**Criminal**				
X9.1	Loss of materials and equipment during project implementation	68	295	4	High influence
X9.2	Damage to tools, materials, and facilities by irresponsible parties	68	296	4	High influence
X9.3	The occurrence of petty corruption practices by project workers	68	297	4	High influence
X9.4	Use of illegal substances and drugs by workers	68	292	4	High influence
X9.5	Extortion from outside	68	305	4	High influence
X9.6	There are fights between workers	68	290	4	High influence
**X10**	**Project Manager**				
X10.1	Poor ability of the project manager in selecting personnel involved in the project.	68	285	5	Very influential
X10.2	Lack of project manager experience in dividing tasks and responsibilities	68	289	5	Very influential
X10.3	Lack of project manager experience in scheduling all work activities	68	297	5	Very influential
X10.4	Lack of project manager communication skills with the owner when coordinating during the project	68	269	4	High influence
X10.5	Poor communication between the project manager and his team, including sub-contractors.	68	263	4	High influence
X10.6	Project managers do not encourage the whole team to work in totality.	68	245	4	High influence
X10.7	Lack of monitoring and control during project implementation	68	295	4	High influence

## Conclusion

Based on the results of the research, it can be concluded that, of the 60 identified risk factors causing project delays, 30 risk indicators are in the *very influential* category with a mode value of (= 5), 29 risk indicators are included in the *high influence* category with a mode value of (= 4), and one indicator is included in the *medium influence* category (= 3). This research contributes to the development of the literature and expands the research related to the theoretical field of risk factors causing project delays. The results of the study provide information for contractors, supervision, consultants, and owners regarding the most influential factors causing delays occurring during the implementation of construction projects in Aceh Province; accordingly, they are expected to provide an overview for managing the risks of uncertainty in the implementation of construction projects and avoiding losses. Moreover, this information can help increase profits while maintaining the specified quality, and successful risk management can be applied to implement projects, to accelerate project work, and to reduce overall costs [42, 43].

Time, quality, and cost are three important components when planning a construction project. The benchmark for a successful construction project is the completion time following the timeframe provided, with minimal costs, and without compromising the quality of construction. Contractors must be able to manage a construction project systematically so that the project completion time follows the contract or even faster so that the costs incurred can be an advantage, and also to avoid fines owing to being late in completing construction projects. Therefore, acceleration is important to overcome delays. This study has limitations, namely, this research was conducted in the Aceh Province, the results of this study are unique and can only be claimed to be true only for the Province of Aceh, Indonesia, and not necessarily true in other regions of Indonesia. Thus, similar research can be conducted in other provinces and cities in Indonesia, so that the results can be generalised to other regions, and the number of samples in this study was 68 respondents. Therefore, it is necessary to increase the number of respondents for further research so that the research results are more comprehensive.
