# Correction: Causes of delays in construction projects in the Province of Aceh, Indonesia

**DOI:** 10.1371/journal.pone.0329775

**Published:** 2025-08-04

**Authors:** Anita Rauzana, Wira Dharma

Table 2 is formatted incorrectly. The decimals in the “Cronbach Alpha” column should be separated by a period not a comma. Please see the correct Table 2 here.

**Table 2:** Reliability test.

No.	Variable	Cronbach Alpha	Conclusion
1	Material (X_1_)	0.943	Reliable
2	Equipment (X_2_)	0.819	Reliable
3	Financial (X_3_)	0.916	Reliable
4	Environment (X_4_)	0.903	Reliable
5	Labour (X_5_)	0.944	Reliable
6	Implementation (X_6_)	0.787	Reliable
7	Management (X_7_)	0.937	Reliable
8	Political (X_8_)	0.638	Reliable
9	Criminal (X_9_)	0.913	Reliable
10	Project Manager (X_10_)	0.902	Reliable
